# Mother–Daughter Vessel Operation and Maintenance Routing Optimization for Offshore Wind Farms Using Restructuring Particle Swarm Optimization

**DOI:** 10.3390/biomimetics9090536

**Published:** 2024-09-05

**Authors:** Yuanhang Qi, Haoyu Luo, Gewen Huang, Peng Hou, Rongsen Jin, Yuhui Luo

**Affiliations:** 1 School of Computer Science, University of Electronic Science and Technology of China, Zhongshan Institute, Zhongshan 528402, China; qiyuanhang@zsc.edu.cn (Y.Q.); haoyuluo22@163.com (H.L.); 2 School of Automation, Guangdong University of Technology, Guangzhou 510006, China; luoyh2020@163.com; 3 Information and Network Center, Jiaying University, Meizhou 514015, China; huang_gewen@163.com; 4 Smart Wind Energy Team, Zhejiang Baima Lake Laboratory Co., Ltd., Hangzhou 310052, China; 5 The Chinese University of Hong Kong (Shenzhen), Shenzhen 518116, China; rongsenjin@link.cuhk.edu.cn

**Keywords:** operation and maintenance routing optimization, restructuring particle swarm optimization, offshore wind farm, mother vessel

## Abstract

As the capacity of individual offshore wind turbines increases, prolonged downtime (due to maintenance or faults) will result in significant economic losses. This necessitates enhancing the efficiency of vessel operation and maintenance (O&M) to reduce O&M costs. Existing research mostly focuses on planning O&M schemes for individual vessels. However, there exists a research gap in the scientific scheduling for state-of-the-art O&M vessels. To bridge this gap, this paper considers the use of an advanced O&M vessel in the O&M process, taking into account the downtime costs associated with wind turbine maintenance and repair incidents. A mathematical model is constructed with the objective of minimizing overall O&M expenditure. Building upon this formulation, this paper introduces a novel restructuring particle swarm optimization which is tailed with a bespoke encoding and decoding strategy, designed to yield an optimized solution that aligns with the intricate demands of the problem at hand. The simulation results indicate that the proposed method can achieve significant savings of 28.85% in O&M costs. The outcomes demonstrate the algorithm’s proficiency in tackling the model efficiently and effectively.

## 1. Introduction

Due to the unceasing alterations in the global climate, the issues of energy supply and cost have ascended as pressing concerns for current governments and societies [1]. In recent years, there have been endeavors to reduce the emissions of carbon dioxide and other pollutants that exacerbate global warming effects [2]. Wind energy, as a sustainable and clean source, has attracted extensive attention [3]. Notably, offshore wind power, owing to its higher energy generation efficacy, has emerged as one of the most rapidly expanding energy sources in recent years [4]. In Europe, the installed capacity of offshore wind farms has achieved a compound annual growth rate of 22% over the past decade. By 2030, it is anticipated that the total installed capacity of offshore wind farms in Scandinavia will reach 45 GW [5,6,7]. Compared to onshore wind power, offshore wind offers fewer spatial constraints, higher capacity benefits, and longer turbine lifespans, prompting designers to deploy turbines of greater capacity to enhance power generation [8]. However, due to the unique operational environment and geographical positioning, offshore wind turbines (WTs) exhibit higher failure rates than their onshore counterparts [9]. As the industry advances, offshore wind farms are increasingly located further from the coast, in deeper waters, and under more severe marine conditions, which can result in significant economic impacts due to extended downtimes during maintenance or repairs. Consequently, offshore wind farms necessitate stringent operational and maintenance standards, incurring significant costs including turbine maintenance, vessel upkeep, and insurance, which typically account for 20% to 35% of the total operational lifecycle costs [10]. Offshore wind farm operations and maintenance encompass strategy selection, schedule optimization, onsite operations, repair, assessment criteria, recycling, and environmental concerns [11], as well as turbine model selection [12]. The overarching goal of these activities is to reduce operation and maintenance (O&M) costs and minimize production losses. Given the large number of turbines in a wind farm and the need for regular maintenance, it is essential to develop maintenance plans that minimize production losses and downtime [13]. In [13], the issue of optimizing vessel fleet size and composition for offshore wind farm maintenance was addressed, focusing on the most cost-effective assignments of vessels to routes and technicians to vessels. Li et al. [14] focused on reducing O&M costs, ensuring environmental protection around the wind farm, and enhancing the safety of the O&M process. They set objectives to minimize total O&M costs, carbon emissions from O&M vessels, and the standard deviation of vessel sailing duration. Ma et al. [15] addressed the challenges of large-scale, multi-vessel cooperative maintenance scheduling in offshore wind farms. In [8], an O&M scheduling methodology for offshore wind farm operations, based on wind and wave forecasts, was implemented, accounting for weather forecast uncertainty. As demonstrated in the aforementioned studies, a critical common issue is the optimization of O&M routes, making operation and maintenance routing optimization (OMRO) a pivotal research direction [16].

To resolve the OMRO problem, it is crucial to consider the actual operational requirements of WTs, which include both corrective repairs and preventive maintenance tasks. Corrective maintenance pertains to the rectification of turbine malfunctions [17]. Lazakis et al. introduced an optimization framework for daily or short-term maintenance operations based on routing planning and scheduling [18]. On the other hand, preventive maintenance encompasses routine inspections and component replacements to pre-empt failures [11]. Liu et al. proposed a model that accounts for the priorities of maintenance tasks and the practical constraints of scheduling [19]. O’Neil et al. developed an integrated maintenance and orientation framework that addresses the selection of WT access, component maintenance, maintenance level execution, repair personnel allocation, and their routing [20]. In real-world applications, there is often a need to perform preventive and corrective maintenance simultaneously. Dai et al. delved into the optimization of maintenance fleet routing and scheduling in offshore wind farms, aiming at optimizing the distribution of turbines and vessel routes with minimal costs [21]. Irawan et al. considered multiple vessels and maintenance bases and developed an optimization model for the maintenance routing and scheduling of offshore wind farms using an Dantzig–Wolfe decomposition-based algorithm [22]. Moreover, considering that the losses from corrective maintenance are calculated from the onset of turbine damage and continue until repairs are completed, Feng et al. categorized the turbine power losses into fault-induced and maintenance-induced losses, aiming to minimize these losses alongside the transportation and technician wage costs [23]. Hadjoudj and Pandit [24] designed a data-driven decision tool to enhance vessel routing for O&M tasks under various environmental conditions, such as wind speed, wave period, and wave height. A framework was proposed in [25] for integrating tactical and operational optimization models for maintenance scheduling and routing in offshore wind farms. Within this framework, an operational maintenance routing model was solved to obtain optimal vessel routes within a one-day planning horizon, utilizing a metaheuristic approach that incorporates variable neighborhood search and an exact method. However, the aforementioned studies typically relied on service operations vessels (SOVs) for maintenance operations, either independently or jointly, which leads to substantial expenses related to the rental, fuel, and operational overheads.

Currently, maritime companies are pioneering the development of specialized SOVs equipped with daughter vessels (DVs) tailored for operations in deep-sea wind farms. These SOVs are equipped with advanced onboard transfer systems that enable the seamless deployment and recovery of DVs, along with the efficient movement of personnel and repair equipment. Leveraging the swift maneuverability and economical operation of DVs, these maritime assets substantially mitigate maintenance expenses. Moreover, SOVs serve as mobile offshore bases, providing accommodations for the crew and storage for equipment, thereby enhancing their suitability for maintenance tasks within deep-sea wind farms. Chandra et al. [26,27] explored maintenance involving a mother vessel with DVs using this type of vessel configuration. However, their study was limited to an analysis of the power losses attributable to turbine maintenance. Consequently, this paper proposes a more comprehensive approach by incorporating both the fault-induced and maintenance-induced power losses. We present an optimization model for mother–daughter vessel operation and maintenance routing for offshore wind farms (MDVOMR), aiming to achieve a holistic optimization of the maintenance process and associated costs. The objectives of this model include minimizing the downtime of malfunctioning turbines and reducing the cost of vessels by employing effective coordination between the SOV and DVs to complete operation and maintenance tasks.

The optimization of routing schedules for offshore wind farms presents a sophisticated combinatorial optimization challenge, a nuanced variant of the classic vehicle routing problem. Numerous scholars have resorted to intelligent algorithms for resolving such an intricate problem. Wang et al. crafted a hybrid optimization algorithm that merges ant colony algorithms with dynamic search techniques [28]. Li et al., beginning from the perspective of modestly increasing operational costs to bolster environmental protection surrounding wind farms and enhance the safety of maintenance operations, introduced the chaotic quantum Harris hawks optimization algorithm, forging a novel approach to optimize maintenance scheduling in offshore wind farms [14]. In light of these contributions, this paper also employs intelligent algorithms to tackle MDVOMR. The algorithm employs an encoding strategy using a vector that combines real and binary numbers, representing the sequence of turbines and the type of transport vehicle, respectively. The decoding strategy utilizes ascending order sorting to determine the service sequence of turbines and a position adjustment method at the endpoints to select the transport vehicle sequence for the turbines. Additionally, the algorithm introduces a segmentation method to determine the service routes for the SOV and DV.

The contributions of this paper are as follows: (1) By leveraging a service operation vessel (SOV) equipped with a daughter vessel (DV), this paper introduces a comprehensive approach that adeptly handles both preventive maintenance and corrective repairs. This methodology, termed MDVOMR, is designed to minimize the downtime of malfunctioning turbines and reduce vessel operational costs. It achieves these objectives through the seamless coordination between the SOV and the DVs. By integrating preventive and corrective maintenance routing with a backhaul scheme, the model not only streamlines the process but also effectively reduces the cost and time associated with unplanned maintenance interventions. (2) Effective encoding and decoding strategies are proposed and integrated into the restructuring particle swarm optimization (RPSO) [29] for solving MDVOMR. The encoding strategy utilizes a vector composed of both real and binary numbers. It incorporates ascending order sorting, a position adjustment method, and a segmentation method to determine the service routes for the SOV and DV, thereby enhancing the algorithm’s search efficiency and quality and accelerating the process of finding the optimal solution. (3) Grounded in a real-world operational scenario involving twelve WTs within an offshore wind farm, the efficacy of the proposed model and algorithm is empirically validated through simulations.

The structure of this paper is as follows: Section 2 introduces the MDVOMR model. Section 3 provides a detailed description of the RPSO algorithm used to solve this model. Section 4 selects a wind farm with 12 WTs as a case study to illustrate the effectiveness of the proposed model and its solution method. Finally, Section 5 presents the conclusions and explores potential directions for future research.

## 2. Mathematical Models

In this section, the model description and assumptions are introduced. A new operation and maintenance routing optimization model is proposed, which is based on a mother vessel with a daughter vessel.

### 2.1. Problem Description

In an organization operating an offshore wind farm, multiple turbines are strategically deployed. Among these, some experience damage that requires prompt attention and repair, while others need routine maintenance to ensure ongoing operational efficiency. A scheduling problem involves creating a multi-day plan to determine which turbines will be maintained on specific days. A routing optimization problem determines the sequence of turbines serviced based on vessel limitations, which this paper addresses. In this scenario, an SOV departs from the port carrying a DV. The SOV functions as a mobile maintenance hub for the DV, providing essential support such as equipment, personnel, and fuel. Upon reaching a turbine, the SOV and DV collaborate to dispatch personnel and equipment to the turbine and subsequently retrieve them. After the retrieval is complete, the SOV returns to the port with the DV.

**Figure 1:**
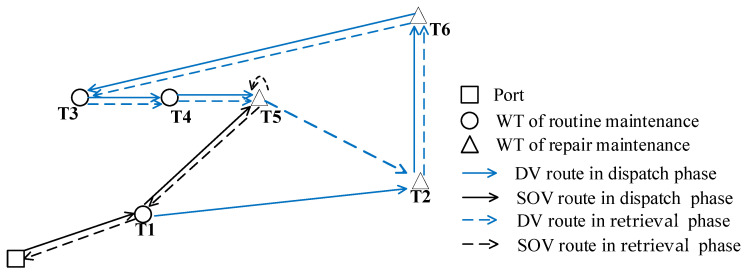
The dispatch and retrieval route schematic.

MDVOMR is a strategic orchestration of the deployment of an SOV equipped with a DV to maintain and repair WTs. This process encompasses both the dispatch and retrieval phases. The initial phase involves the SOV departing from the port and dispatching personnel to the WT units for maintenance activities. The subsequent phase entails the retrieval of both personnel and equipment after the completion of maintenance or repair activities, marking the vessel’s return journey to port. The objective of MDVOMR is to meticulously craft dispatch and retrieval schemes of the vessel, considering the comprehensive costs of power losses, equipment transfer, and the leasing and fuel expenses associated with the SOV and DV, with a target to minimize total costs. It can be seen that MDVOMR is a generalized instance of the vehicle routing problem of backhauls. The operational schematic of MDVOMR, as illustrated in Figure 1, delineates emergency repairs in turbines marked by triangles and routine maintenance in turbines indicated by circles. Solid lines represent the dispatch routes, and dashed lines depict retrieval routes. This schematic exemplifies a modest operational event involving maintenance tasks across six turbines, two of which are out of operation, requiring immediate corrective repairs, and four are scheduled for preventive maintenance.

This model is designed to orchestrate an optimal operational route for the SOV and DV, considering the cumulative power generation losses that the wind farm operator may face and the vessel usage costs associated with wind power maintenance. By doing so, this model strikes a balance between minimizing downtime and operational expenses. It is adept at handling scenarios that involve either self-maintenance by the wind farm or third-party maintenance services, where an SOV is utilized to transport and support a DV. This model’s versatility makes it a valuable tool for enhancing the efficiency and cost-effectiveness of offshore wind farm maintenance operations.

### 2.2. Mathematical Model

This section initially delineates the meanings of the symbols employed in the optimization model, followed by the formulation of the objective function based on the model’s aims. Lastly, the constraints within the model are explicated through constraint functions.

#### 2.2.1. Mathematical Variables

Sets

$I^{-}$: The set of WTs requiring service during the dispatch phase, $I^{-}=\{1,\ldots ,n\}$, and $i^{-}\in I^{-}$;

$I^{+}$: The set of WTs requiring service during the retrieval phase, $I^{+}=\{n+1,\ldots ,2n\}$; The elements of set $\hat{I}$ correspond one to one with the elements of set $I^{-}$, and $i^{+}\in I^{+}$;

I: The set of WTs requiring service during the dispatch and retrieval phase, I={1,…,2n}, and $i,i^{\prime }\in I$;

U: The set of turbines requiring urgent maintenance and $U\subseteq I^{-}$;

N: The set of total nodes, and N={0,…,2n+1}, where nodes 0 and 2n+1 represent the ports where the vessel’s route begins and ends, respectively;

L: The maximum voyage count of the DV, and *l* represents the number of voyages, l∈{1,…,L}.

Constants

$D_{\max}$: The maximum sailing distance of the DV;

$W_{\max}$: The maximum load capacity of the DV;

$V^{\mathrm{DV}}$: The cruising speed of the DV;

$V^{\mathrm{SOV}}$: The cruising speed of the SOV;

$C^{\mathrm{DV}}$: The operational cost per hour for the DV;

$C^{\mathrm{SOV}}$: The operational cost per hour for the SOV;

${\tilde{C}}^{\mathrm{DV}}$: The docking cost per hour for the DV;

${\tilde{C}}^{\mathrm{SOV}}$: The docking cost per hour for the SOV;

$E_{L}$: The economic loss per hour during turbine downtime;

$T_{\mathrm{UD}}$: The personnel and equipment transfer time between the DV and SOV, and the DV refueling time is also included.

Variables

$\tau_{i}^{\mathrm{LD}}$: Time required for SOV/DV to transfer equipment at turbine *i*;

$w_{i}$: Total weight required for preventive maintenance or corrective repairs on turbine *i*;

$\tau_{i}$: Time required to perform preventive maintenance or corrective repairs on turbine *i*;

$d_{ij}$: Distance from node *i* to node *j*.

Decision Variables

$$s_{ij}=\{\begin{array}{cc} 1 & \mathrm{The}\;\mathrm{SOV}\;\mathrm{sails}\;\mathrm{from}\;\mathrm{node}i\;\mathrm{to}\;\mathrm{node}\;j\\ 0 & \mathrm{Otherwise} \end{array}$$

$$u_{i}=\{\begin{array}{cc} 1 & \mathrm{The}\;\mathrm{SOV}\;\mathrm{docks}\;\mathrm{at}\;\mathrm{turbine}\;i\mathrm{to}\;\mathrm{render}\;\mathrm{service}\\ 0 & \mathrm{Otherwise} \end{array}$$

$$y_{ijl}=\{\begin{array}{cc} 1 & \;\mathrm{The}\;\mathrm{voyage}\;l\;\mathrm{of}\;\mathrm{the}\;\mathrm{DV}\;\mathrm{is}\;\mathrm{from}\;\mathrm{node}\;i\;\mathrm{to}\;\mathrm{node}\;j\\ 0 & \mathrm{Otherwise} \end{array}$$

$$z_{il}=\{\begin{array}{cc} 1 & \mathrm{The}\;\mathrm{DV}\;\mathrm{docks}\;\mathrm{at}\;\mathrm{turbine}\;i\mathrm{to}\;\mathrm{provide}\;\mathrm{service}\;\mathrm{during}\;\mathrm{voyage}\;l\;\\ 0 & \mathrm{Otherwise} \end{array}$$

$$o_{il}=\{\begin{array}{cc} 1 & \mathrm{The}\;\mathrm{SOV}\;\mathrm{and}\;\mathrm{the}\;\mathrm{DV}\;\mathrm{separate}\;\mathrm{at}\;\mathrm{turbine}i\;\mathrm{during}\;\mathrm{voyage}\;l\;\\ 0 & \mathrm{Otherwise} \end{array}$$

$$q_{il}=\{\begin{array}{cc} 1 & \mathrm{The}\;\mathrm{DV}\;\mathrm{returns}\;\mathrm{to}\;\mathrm{the}\;\mathrm{SOV}\;\mathrm{at}\;\mathrm{turbine}i\;\mathrm{during}\;\mathrm{voyage}\;l\;\\ 0 & \mathrm{Otherwise} \end{array}$$

$\tau_{i}^{\mathrm{wait}}$: The waiting time for corrective maintenance for damaged turbine *i*;

$T_{i}^{\mathrm{SOV}}$: The cumulative time taken for the SOV to reach turbine *i*;

$T_{il}^{\mathrm{DV}}$: The cumulative time taken for the DV to reach turbine *i* during voyage *l*;

$P\tau_{i}^{\mathrm{SOV}}$: The docking time of the SOV at turbine *i*;

$P\tau_{il}^{\mathrm{DV}}$: The time that the DV docks at turbine *i* during voyage *l*;

$W_{il}^{\mathrm{DV}}$: The cumulative load of the DV when departing from turbine *i* during voyage *l*;

$D_{il}^{\mathrm{DV}}$: The cumulative travel distance of the DV when leaving turbine *i* during voyage *l*.

#### 2.2.2. Objective Function

The objective of the model is to minimize the total operational and maintenance costs. The overall cost includes electricity generation losses (EGLs), equipment transfer costs, and the leasing and fuel expenses associated with the SOV and DV. The EGL is linearly related to the waiting time and actual maintenance time. The leasing and fuel expenses associated with the SOV and DV can be converted into costs related to docking time and traveling distance, while equipment transfer operations incur additional docking time. Therefore, the overall cost includes expenses incurred from the waiting time, actual maintenance time, docking time, and traveling distance, as demonstrated in Equation (1).

(1) $$\begin{array}{cc} \min z= & E_{L}(\sum_{i_{D}\in U}\tau_{i_{D}}^{\mathrm{wait}}+\sum_{i^{-}\in I^{-}}\tau_{i^{-}})\\ & +\frac{C^{\mathrm{SOV}}}{V^{\mathrm{SOV}}}\sum_{i\in N}\sum_{j\in N}s_{ij}d_{ij}+{\tilde{C}}^{\mathrm{SOV}}\sum_{p\in P}P\tau_{i}^{\mathrm{SOV}}\\ & +\frac{C^{\mathrm{DV}}}{V^{\mathrm{DV}}}(\sum_{i\in N}\sum_{j\in N}\sum_{l=1}^{L}y_{ijl}d_{ij})+{\tilde{C}}^{\mathrm{DV}}\sum_{i\in I}\sum_{l=1}^{L}P\tau_{il}^{\mathrm{DV}} \end{array}$$

The first part of the formula represents the loss costs due to turbine downtime, denoted as EGL costs; the second part encompasses the operational costs of the SOV; the third part includes the docking costs of the SOV; the fourth part covers the operational costs of the DV; and the fifth part accounts for the docking costs of the DV. Notably, both EGL costs and vessel costs exhibit a linear correlation with the passage of time.

#### 2.2.3. Problem Formulation

During the operational and maintenance processes of the SOV and the DV, both vessels navigate at a constant speed, with the distance between points calculated as the Euclidean distance. Notably, the first turbine reached during the dispatch phase and the last turbine serviced in the retrieval phase require the SOV’s intervention. Upon completing the dispatch phase, the SOV remains at the last turbine to await the return of all DVs before commencing the retrieval phase tasks. Consequently, there are three distinct nodes: the initial turbine reached at the start, the transitional node between the dispatch and retrieval phases, and the final node where the SOV docks during the retrieval phase. Each of these special nodes is subject to specific constraints which will be specified in the following.

- As the vessels depart from the port, Constraints (2)–(4) stipulate that the SOV, carrying the DV, sets out from the port to initiate service at the first turbine. It is imperative that the DV does not embark directly from the port. Constraint (5) specifies that the cumulative time is initialized to zero at the SOV’s departure from the port, with the elapsed time to the first turbine being determined by the transit time from the port to the turbine’s location.
  (2) $$\sum_{\tilde{i}\in \tilde{I}}s_{0i^{-}}=1$$
  (3) $$\sum_{i^{-}\in I^{-}}\sum_{l=1}^{L}y_{0i^{-}l}=0$$
  (4) $$u_{i^{-}}-M(1-s_{0i^{-}})\geq 1$$
  (5) $$T_{i}^{\mathrm{SOV}}\geq \frac{d_{0i}}{V^{\mathrm{DV}}}-M(1-s_{0i})$$
- In this work, we establish a protocol where dispatch tasks must be executed before retrieval tasks begin. It is assumed that *I^−^* represents the set of WTs requiring service during the dispatch phase, while $I^{+}$ represents those requiring service during the retrieval phase. When the vessels complete the dispatch phase, the SOV docks at the last-serviced turbine until the DV returns. Then, the retrieval phase begins, and the SOV travels to the first-serviced turbine, carrying the DV. Thus, only the SOV travels from one of the turbines in $I^{-}$ to one in set $I^{+}$, while the DV does not. Neither vessel can travel from any turbine in $I^{+}$ to any turbine in $I^{-}$. Constraint (6) indicates that the SOV will proceed directly from the last-serviced turbine in the dispatch phase to the first turbine in the retrieval phase without deviation. Constraint (7) delineates that the DV is restricted from traversing between the dispatch and retrieval phases. Constraint (8) dictates that neither the SOV nor the DV is allowed to revisit the turbines from the dispatch phase once the retrieval phase has begun.
  (6) $$\sum_{i^{-}\in I^{-}}\sum_{i^{+}\in I^{+}}s_{i^{-}i^{+}}=1$$
  (7) $$\sum_{i^{-}\in I^{-}}\sum_{i^{+}\in I^{+}}\sum_{l=1}^{L}y_{i^{-}i^{+}l}=0$$
  (8) $$\sum_{i^{-}\in I^{-}}\sum_{i^{+}\in I^{+}}\left(s_{i^{-}i^{+}}+\sum_{l=1}^{L}y_{i^{-}i^{+}l}\right)=0$$
- Upon the completion of all tasks and the vessels’ return to port, Constraints (9)–(11) dictate that the SOV, after servicing the final turbine, must return to the port with all DVs embarked. This regulation precludes the DVs from making any independent return journeys, ensuring a coordinated and unified operation for the return lag of the mission.
  (9) $$\sum_{i^{+}\in I^{+}}s_{i^{+},2n+1}=1$$
  (10) $$\sum_{i^{+}\in I^{+}}\sum_{l=1}^{L}y_{i^{+},2n+1,l}=0$$
  (11) $$u_{i^{+}}-M(1-s_{i^{+},2n+1})\geq 1$$

To ensure continuity, it is imperative to implement constraints that govern the separation of the DVs from the SOV at the serviced turbines, as well as for the return of the DVs to the SOV at the turbines serviced by the SOV. These constraints will be elaborated in the following.

- In this work, the DVs must both detach from and reconnect with the SOV at designated turbine sites, precluding the possibility of mid-route reconnections. The turbines identified for separation inherently become the responsibility of the SOV, thus imposing specific operational constraints for the processes of detachment and reconnection. Constraints (12) and (13) indicate that when the SOV is docked at a serviced turbine, it is permitted to execute the operations of separation and reconnection with the DV. Constraints (14) and (15) define the criteria for determining the reconnection and separation points along each leg of the DV’s itinerary. Constraint (16) specifies that the SOV will remain in wait for the DV’s return at the subsequent turbine designated for service. Constraint (17) mandates that the DV must service at least one turbine before its return following detachment. Constraints (18)–(21) stipulate that each voyage of the DV includes only one reconnection and one separation point, and these points must not overlap within the same phase for successive voyages, ensuring a seamless and efficient operational flow.
  (12) $$u_{i}\geq \sum_{l=1}^{L}o_{il}$$
  (13) $$u_{i}\geq \sum_{l=1}^{L}q_{il}$$
  (14) $$\sum_{j\in U}y_{ijl}\sum_{j\in U}s_{ij}=o_{il}$$
  (15) $$\sum_{j\in U}y_{jil}\sum_{j\in U}s_{ji}=q_{il}$$
  (16) $$o_{\bar{i}l}\geq 1-M(1-s_{i\bar{i}}o_{il}),i\neq \bar{i}$$
  (17) $$z_{\bar{i}l}+M(1-o_{il}y_{i\bar{i}l})\geq 1$$
  (18) $$\sum_{i\in I}o_{il}\leq 1$$
  (19) $$\sum_{i\in I}r_{il}\leq 1$$
  (20) $$(1-o_{il})(1-o_{i\bar{l}})=0,l\neq \bar{l}$$
  (21) $$(1-r_{il})(1-q_{i\bar{l}})=0,l\neq \bar{l}$$
- Considering the operational constraints of the DV, which include the maximum load capacity and a stipulated travel distance, it is imperative to regulate the cumulative load and journey distance for DVs at each turbine. Constraints (22) and (23) address the capacity limitations of the DVs, while Constraints (24) and (25) govern the travel distances.
  (22) $$W_{\bar{i}l}^{\mathrm{DV}}\geq W_{il}^{\mathrm{DV}}+w_{\bar{i}}z_{\bar{i}l}-M(1-y_{i\bar{i}l}),i\neq \bar{i}$$
  (23) $$W_{il}^{\mathrm{DV}}\leq W_{\max}$$
  (24) $$D_{\bar{i}l}^{\mathrm{DV}}\geq D_{il}^{\mathrm{DV}}+d_{i\bar{i}}-M(1-y_{i\bar{i}l})$$
  (25) $$D_{il}^{\mathrm{DV}}\leq D_{\max}$$
- In the maintenance operations at each turbine node, a single vessel is sufficient to fulfill the tasks. Thus, it is critical to maintain a balance between the arrival and departure of vessels at these nodes. Constraint (26) specifies that each turbine can be serviced by only one vessel within any given phase, thereby preventing any overlap in service assignments. Furthermore, Constraints (27) and (28) ensure that the servicing of each turbine by any vessel maintains a balance between vessel arrivals and departures.
  (26) $$u_{i}+\sum_{l=1}^{L}z_{il}=1$$
  (27) $$\sum_{\bar{i}\in I}z_{i\bar{i}}=\sum_{\bar{i}\in I}z_{\bar{i}i}=u_{i}$$
  (28) $$(1-o_{il})(1-r_{il})\sum_{\bar{i}\in I}y_{i\bar{i}l}=(1-o_{il})(1-q_{il})\sum_{\bar{i}\in I}y_{\bar{i}il}=(1-o_{il})(1-q_{il})z_{il}$$

In this work, the objective function is closely related to time; a series of meticulous constraints are delineated to encompass the total time expenditure for both the SOV and the DV, as well as the waiting time caused by the turbine maintenance, equipment transfers, and the vessel’s early arrival at reunion points. Specifically, the personnel and equipment loading and unloading times at each turbine for the SOV/DV are set at distinct values, and the transfer operation times for DV separation and reunion with the SOV are constants.

To record the time of each turbines waiting the SOV or the DV’s arriving and precisely calculate the objective function, it is imperative to establish constraints that govern the accumulated time for vessel services and travel. Constraint (29) outlines the total time consumed by the SOV in proceeding to the subsequent turbine, encompassing the departure time from the preceding turbine, the cumulative service and docking duration at the prior turbine, and the transit time between the two locations. Constraint (30) indicates that the cumulative time for the DV, when departing from the SOV, should not be less than that of the SOV. Constraint (31) indicates that when the DV immediately departs from the SOV after reloading and resupplying from a previous journey, its cumulative time should include the SOV’s cumulative time and transfer time between the DV and SOV. Similarly, the total duration for the DV to reach the subsequent turbine, incorporating the departure time from the previous turbine, the service and docking time there, and the travel time between them, is described in (32).

(29) $$T_{\bar{i}}^{\mathrm{SOV}}\geq T_{i}^{\mathrm{SOV}}+P\tau_{i}^{\mathrm{SOV}}+\frac{d_{i\bar{i}}}{V^{\mathrm{SOV}}}-M(1-s_{i\bar{i}}),i\neq \bar{i}$$

(30) $$T_{il}^{\mathrm{DV}}\geq T_{i}^{\mathrm{SOV}}-M(1-o_{il})$$

(31) $$T_{il}^{\mathrm{DV}}\geq T_{i}^{\mathrm{SOV}}+T_{\mathrm{UD}}-M(1-o_{il})-M(1-\sum_{l=1}^{L}q_{il})$$

(32) $$T_{\bar{i}l}^{\mathrm{DV}}\geq T_{il}^{\mathrm{DV}}+P\tau_{il}^{\mathrm{DV}}+\frac{d_{i\bar{i}}}{V^{\mathrm{DV}}}-M(1-y_{i\bar{i}l}),i\neq \bar{i}$$

- Given the additional waiting times incurred by servicing turbines, transferring equipment, and vessels arriving early at rendezvous points, detailed constraints for these intervals are also necessary. Equations (33) and (34) stipulate that the docking durations at turbines serviced by the DV and SOV must at least cover the time required to transfer equipment at those turbines. Equations (35) and (36) specify that at separation points, the docking times for the DV and SOV must satisfy the requirements for completing equipment transfers between them, including the time for DV refueling. Equation (37) dictates that the docking time for the DV at rendezvous points must include the time waiting for the SOV to arrive and the time to transfer equipment to the SOV. Equation (38) indicates that at rendezvous points, the SOV’s docking time, while engaging in turbine equipment transfer and DV equipment transfer operations, must also encompass the additional time potentially required due to waiting for the DV’s arrival. Equation (39) accounts for the scenario where the DV immediately commences a new voyage after completing a previous segment, considering two successive equipment transfers between the DV and SOV based on Equation (38). Equations (40) and (41) state that during the retrieval phase, the SOV or DV, upon arriving at a turbine, may need to wait for the completion of maintenance/repair works on that turbine before beginning loading or unloading operations, thus adding extra waiting time.
  (33) $$P\tau_{il}^{\mathrm{DV}}\geq \tau_{i}^{\mathrm{LD}}-M(1-z_{il})$$
  (34) $$P\tau_{i}^{\mathrm{SOV}}\geq \tau_{i}^{\mathrm{LD}}-M(1-u_{i})$$
  (35) $$P\tau_{il}^{\mathrm{DV}}\geq T_{\mathrm{UD}}-M(1-o_{il})$$
  (36) $$P\tau_{i}^{\mathrm{SOV}}\geq T_{\mathrm{UD}}-M(1-\sum_{l=1}^{L}o_{il})$$
  (37) $$P\tau_{il}^{\mathrm{DV}}\geq \max(T_{i}^{\mathrm{SOV}}-T_{il}^{\mathrm{DV}},0)+T_{\mathrm{UD}}-M(1-q_{il})$$
  (38) $$P\tau_{i}^{\mathrm{SOV}}\geq \max((T_{il}^{\mathrm{DV}}+T_{\mathrm{UD}})-(T_{i}^{\mathrm{SOV}}+\tau_{i}^{\mathrm{LD}}),0)+\tau_{i}^{\mathrm{LD}}-M(1-q_{il})$$
  (39) $$P\tau_{i}^{\mathrm{SOV}}\geq \max((T_{il}^{\mathrm{DV}}+2T_{\mathrm{UD}})-(T_{i}^{\mathrm{SOV}}+\tau_{i}^{\mathrm{LD}}),0)+\tau_{i}^{\mathrm{LD}}-M(1-o_{il})-M(1-\sum_{l=1}^{L}q_{il})$$
  (40) $$\begin{array}{ll} P\tau_{i+n}^{\mathrm{SOV}}\geq & \max(\tau_{i}-((u_{i+n}T_{i+n}^{\mathrm{SOV}}+\sum_{l=1}^{L}z_{i+n,l}T_{i+n,l}^{\mathrm{DV}})-(u_{i}T_{i}^{\mathrm{SOV}}+\sum_{l=1}^{L}z_{il}T_{il}^{\mathrm{DV}})-\tau_{i}^{\mathrm{LD}}),0)+\\ & \tau_{i}^{\mathrm{LD}}-M(1-u_{i}) \end{array}$$
  (41) $$\begin{array}{ll} P\tau_{i+n,l}^{\mathrm{DV}}\geq & \max(\tau_{i}-((u_{i+n}T_{i+n}^{\mathrm{SOV}}+\sum_{l=1}^{L}z_{i+n,l}T_{i+n,l}^{\mathrm{DV}})-(u_{i}T_{i}^{\mathrm{SOV}}+\sum_{l=1}^{L}z_{il}T_{il}^{\mathrm{DV}})-\tau_{i}^{\mathrm{LD}}),0)\\ & +\tau_{i}^{\mathrm{LD}}-M(1-z_{i+n,l}) \end{array}$$

## 3. The Proposed Algorithm

In this section, the RPSO algorithm used in this study is introduced. Subsequently, based on the characteristics of MDVOMR and RPSO, an appropriate encoding and decoding strategy is developed to address the problem.

### 3.1. Restructuring Particle Swarm Optimization

Particle swarm optimization (PSO) is a stochastic optimization technique first proposed by Eberhart and Kennedy in 1995 [30], inspired by the social behavior of bird flocks. In PSO, the formulas for updating particle velocity and position are as follows:

(42) $$v_{i(t+1)}=\omega v_{it}+c_{1}r_{1}\left(pbest_{it}-x_{it}\right)+c_{2}r_{2}\left(gbest_{it}-x_{it}\right)$$

(43) $$x_{i(t+1)}=x_{it}+v_{it}$$

where $v_{it}$ and $v_{i(t+1)}$ denote the velocities of particle *i* at generations t and t + 1, respectively, while $x_{it}$ and $x_{i(t+1)}$ represent the position of particle *i* from generation t to t + 1. $pbest_{it}$ refers to the personal historical best solution of particle *i* at generation *t*, and $gbest_{it}$ signifies the current optimal solution across all particles. ω acts as the inertia factor, $c_{1}$ and $c_{2}$ are the learning factors, and $r_{1}$ and $r_{2}$ are random numbers ranging between 0 and 1.

It is notable that standard PSO often experiences premature convergence and can become trapped in local optima when solving complex optimization problems, limitations that curtail the utility of the PSO algorithm. Given that most enhancement strategies involve replacing the learning model or adjusting parameters to enhance the performance of the PSO algorithm, this results in increased complexity. Zhu and others [29], drawing on linear system theory, proposed the RPSO framework. In RPSO, a singular formula for updating position replaces the traditional formulas for updating both position and velocity.

Assuming that the particle’s own historical best $pbest_{it}$ and the overall best $gbest_{it}$ are constants, the randomness of the system is eliminated. By analyzing the convergence behavior of the swarm through linear system theory and similarly simplifying the PSO system, the trajectory formula for particle position $x_{t}$ during the iterative process is derived.

(44) $$x_{t}=\lambda_{1}+\lambda_{2}\alpha_{t}+\lambda_{3}\beta_{t}$$

Herein, $\lambda_{1}$ represents the cognitive and learning components; $\lambda_{2}\alpha_{t}+\lambda_{3}\beta_{t}$ signifies disturbances. Since a particle’s self-cognition inherently relies on its own historical best position, and the global historical best position forms the basis for social learning, RPSO sets $\lambda_{1}$ as follows:

(45) $$\lambda_{1}=(1-a)\cdot pbest_{it}+a\cdot gbest_{it}$$

Further setting $\lambda_{2}\alpha_{t}+\lambda_{3}\beta_{t}=\delta$ derives the position update formula for RPSO, illustrating the update of position for the *i*-th particle in the *t*-th generation of RPSO:

(46) $$x_{i(t+1)}=(1-a)\cdot pbest_{it}+a\cdot gbest_{it}+\delta$$

Herein, a represents a random number between 0 and 1, and δ denotes random perturbation that decreases with the number of iterations, ensuring that $\underset{t->T}{\lim}\delta =0$. Zhu et al. demonstrated the problem-solving capability of RPSO in [29], while [31] highlights that PSO is highly effective in solving nonlinear functions within multidimensional spaces. Consequently, this paper selects RPSO as the core algorithm for this study.

### 3.2. Encoding and Decoding Strategies

Encoding and decoding can transform real-world problems into formats manageable by algorithms, effectively enhancing the search efficiency and quality of solutions, reducing the search space, and increasing problem-solving efficacy. Building upon the characteristics of the model and RPSO, this section proposes a rapid and effective encoding and decoding strategy, elaborated through a specific example that details the entire encoding and decoding process.

#### 3.2.1. Encoding Strategy

**Figure 2:**
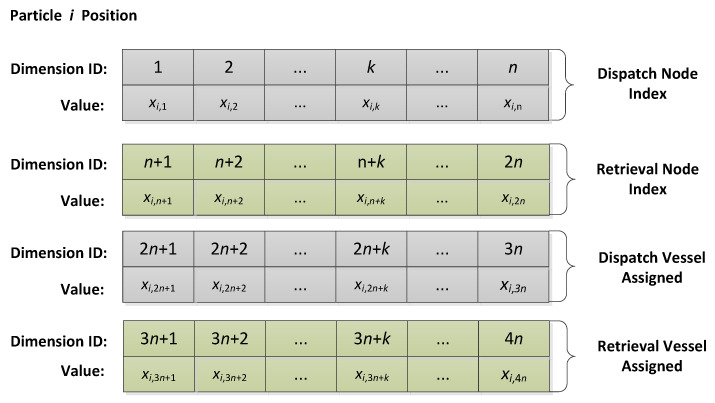
The significance of each dimension of the particle position vector.

According to the model described in Section 2, feasible solutions must encompass sequences of turbines and choices of transportation vehicles for both the dispatch and retrieval phases. Assuming the number of turbines under maintenance is *n*, with *k* as the index, the model assigns sequences for the turbines during both the dispatch and retrieval phases, as well as sequences for transportation vehicle choices in these phases. The position vector of particle *i*, $x_{i}=(x_{i,1},x_{i,2},\ldots ,x_{i,k},\ldots ,x_{i,4n})$, is graphically represented in Figure 2, providing a visual schematic of the model’s configuration. This visualization aids in comprehending the intricate interplay between the sequence of turbine visits and the transportation vehicle assignments, ensuring that the proposed solutions are both coherent and actionable within the operational parameters set forth by the model.

The particle position vector consists of four segments, as follows:

- $x_{i,1},\cdots ,x_{i,n}$ represent the indices of turbines during the dispatch phases, $x_{i,1},\cdots ,x_{i,n}\in [-100,100]$.
- $x_{i,n+1},\cdots ,x_{i,2n}$ represent the indices of turbines during the retrieval phases, $x_{i,n+1},\cdots ,x_{i,2n}\in [-100,100]$.
- $x_{i,2n+1},\cdots ,x_{i,3n}$ correspond to the transportation vehicles assigned to each turbine during the dispatch phases, with $x_{i,2n+1},\cdots ,x_{i,3n}\in \{0,1\}$ denoting the vehicle, where 0 represents the SOV, and 1 represents the DV.
- $x_{i,3n+1},\cdots ,x_{i,4n}$ correspond to the transportation vehicles assigned to each turbine during the retrieval phases, with $x_{i,3n+1},\cdots ,x_{i,4n}\in \{0,1\}$ denoting the vehicle, where 0 represents the SOV, and 1 represents the DV.

**Figure 3:**
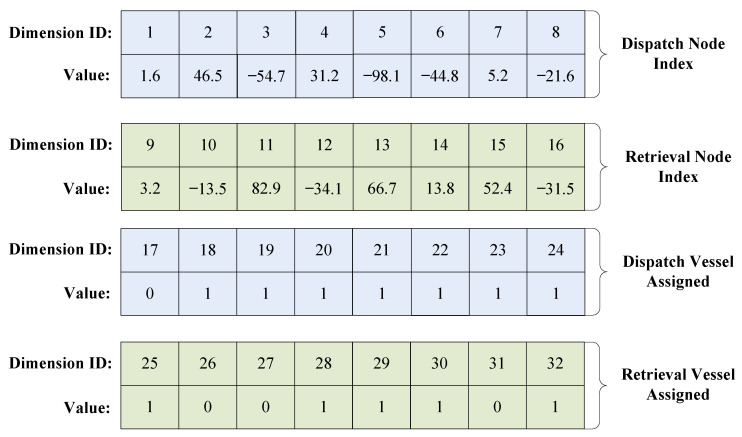
An example of the encoding of a particle’s position vector.

Suppose maintenance involves eight turbines, i.e., *n* = 8; the information spans across 32 dimensions. An example of the position vector encoding of particle *i* is shown in Figure 3. This example will be used to detail the decoding strategy.

#### 3.2.2. Decoding Strategy

Since the decoding processes for both the dispatch and retrieval phases are similar except for differences at the start and endpoints, this section will first detail the decoding process for the dispatch phase, followed by an analogous description for the retrieval phase.

**Figure 4:**
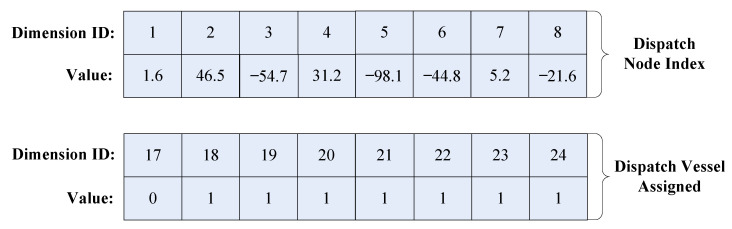
An example of the decoding for the dispatch phase.

Firstly, the decoding process for the dispatch phase is described. As described in the encoding example shown in Figure 3, the sequence of turbines for the dispatch phase (k∈[1,8]) and the sequence for transportation vehicle choices (k∈[17,24]) are displayed in Figure 4.

Next, through three steps, the total service route information for the SOV and DV during the dispatch phase is obtained.

STEP1: The values are sorted in the Dispatch Node Index in ascending order to obtain the corresponding turbine sequence $P_{di}=(5,3,6,8,1,7,4,2)$. The Dimension ID sequence numbers in Dispatch Vessel Assigned are converted by subtracting 2*n* to align with the Dimension ID sequence numbers in the Dispatch Node Index. Then, the values in Dispatch Vessel Assigned are sorted based on the ascending order of the Dispatch Node Index to achieve the corresponding service tool sequence $R_{di}=(1,1,1,1,0,1,1,1)$, yielding the temporary service route information $X_{di}^{Tem}=\{(5,1),(3,1),(6,1),(8,1),(1,0),(7,1),(4,1),(2,1)\}$. Due to the model’s stipulations, the first and last turbines in the dispatch phase must be accessed by the SOV only, thus necessitating a mandatory modification of the service tools for the terminal nodes (5, 1), (1, 1) to 0. Then, $X_{di}=\{(5,0),(3,1),(6,1),(8,1),(1,0),(7,1),(4,1),(2,0)\}$ can be obtained after modification.

STEP2: The nodes serviced by the SOV during the dispatch phase are extracted from $X_{di}$, resulting in $X_{di}^{Sov}=\{(5,0),(1,0),(2,0)\}$. Additionally, the model stipulates that the dispatch phase begins with the SOV carrying the DV from the port; therefore, the port’s starting point (0,0) should be added to the dispatch route, defining the service route of the SOV as $X_{di}^{Sov}=\{\left(0,0\right),\left(5,0\right),\left(1,0\right),\left(2,0\right)\}$.

**Figure 5:**
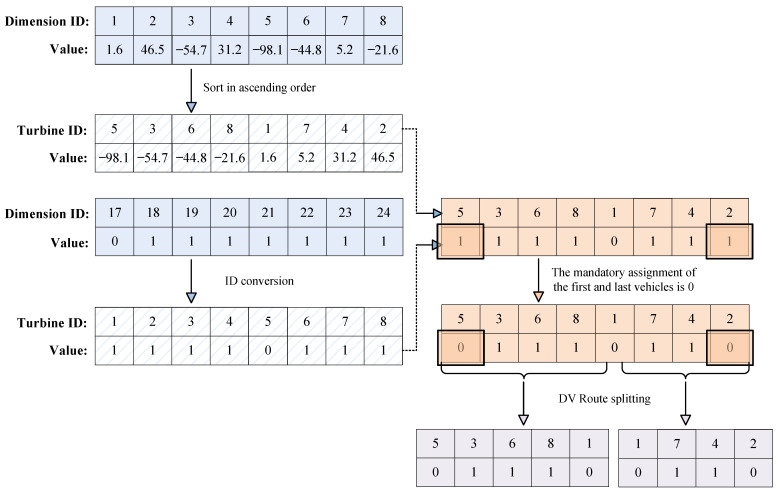
Process of obtaining dispatch phase service routes for SOV and DV.

STEP3: Within the model, the DV can return to the SOV for refueling and equipment transfer. Hence, nodes with a service tool of 0, excluding the initial and final (0,0),(2,0), are designated as segmentation points. The DV must return to these segmentation points at the end of the dispatch phase to complete refueling and equipment replenishment before departing. Segmenting x(i) at points where the service node is 0 yields two routes: Route 1 $X_{di}^{Dv1}=\{(5,0),(3,1),(6,1),(8,1),(1,0)\}$ and Route 2 $X_{di}^{Dv2}=\{(1,0),(7,1),(4,1),(2,0)\}$. The process is illustrated in Figure 5.

The decoding of the retrieval phase is similar to that of the dispatch phase. Firstly, the turbine sequence and transportation vehicle choice sequence are extracted from the encoding example shown in Figure 3 for the retrieval phase, specifically the information between k∈[9,16] and k∈[25,32]. The encoding rules identify nodes 9 to 16 as turbines 1 to 8. The total service routes for the SOV and DV during the retrieval phase are obtained through three steps.

STEP4: Similar to STEP1, the modified total retrieval route information is obtained as $X_{ri}=\{(12,1),(16,1),(10,0),(9,1),(14,1),(15,0),(13,1),(11,0)\}$.

STEP5: The nodes serviced by the SOV are extracted from $X_{ri}$, resulting in $X_{ri}^{Sov}=\{(10,0),(15,0),(11,0)\}$. Moreover, the model dictates that the retrieval phase involves the SOV carrying the DV back to the port, necessitating the addition of the port’s endpoint (11, 0) to the retrieval route, and the first docking point of the SOV during the retrieval phase (10,0) is the endpoint of the dispatch phase (2,0), indicating that the SOV waits in place for the maintenance to conclude. Therefore, the service route of the SOV is defined as $X_{ri}^{Sov}=\{\left(10,0\right),\left(10,0\right),\left(15,0\right),\left(11,0\right),\left(17,0\right)\}$.

STEP6: Segmenting $X_{ri}$ at nodes where the service point is 0 results in three routes. Additionally, since the starting point of the first segment of the DV route is the last stopping point of the SOV dispatch route, (1, 0) must be added before the first segment of the DV route, which is the endpoint of the dispatch phase (2, 0). Route 1: $X_{ri}^{Dv1}=\{(10,0),(12,1),(16,1),(10,0)\}$; Route 2: $X_{ri}^{Dv2}=\{(10,0),(9,1),(14,1),(15,0)\}$; Route 3: $X_{ri}^{Dv3}=\{(15,0),(13,1),(11,0)\}$.

### 3.3. Algorithm Flow

**Figure 6:**
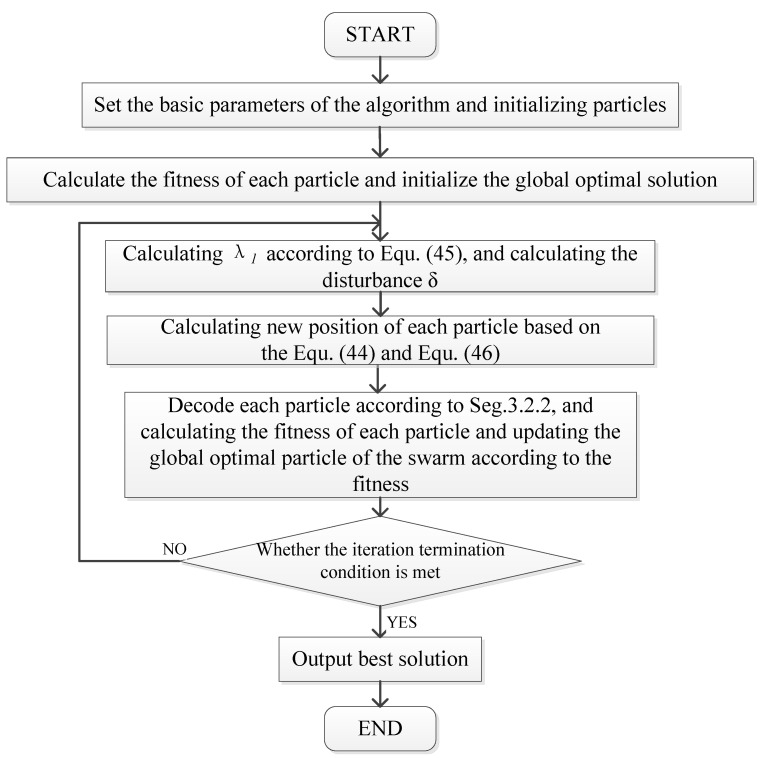
Algorithm flow diagram.

Figure 6 illustrates the algorithmic process: Initially, foundational algorithmic parameters are established, such as swarm size, iteration count, inertia weight, and learning factors; then, particles are initialized, and initial solutions are constructed, evaluating each particle’s fitness and identifying the global optimum particle. Subsequently, the algorithm dynamically refines the inertia weight, the learning factors, and other pertinent parameters through the implementation of the enhanced strategy for exploration. Additionally, the fitness of each particle is calculated, and the global optimal particle of the swarm is updated according to the fitness. Finally, the termination condition is determined, where if it is true, the optimal solution is output; otherwise, the next cycle begins. Due to the strong convergence performance of our algorithm, the termination condition of our algorithm is set to reaching 500 iterations.

## 4. Experiment and Analysis

### 4.1. Experimental Cases and Simulation Environment

The experimental dataset utilizes wind farm site locations from reference [32] and turbine maintenance data from reference [33] as foundational examples. The wind farm points in the experimental dataset were selected from the actual locations of the offshore wind farm (Thanet) owned by Vattenfall, as described in reference [32], and the turbine maintenance time and weight requirements from reference [33] were applied to these selected points. Subsequently, two turbines were randomly selected as those requiring corrective maintenance in this study, and their maintenance time was adjusted to 5 h to ensure adequate servicing. To enhance the realism of our model, actual operational parameters for both SOV and DV were incorporated from references [14,20], with overall example parameters detailed in Table 1. The simulation environment was configured on a Windows 11 platform, powered by an Intel Core i5-11400 CPU @ 2.60 GHz (Intel Corporation, Chandler, AZ, USA), with 32 GB RAM. In the interest of maintaining equitable conditions for analysis, the population size for each algorithm was set at 100, with each running through 500 iterations. To achieve robust and reliable results, each algorithm was run independently ten times.

### 4.2. Experimental Results and Analysis

#### 4.2.1. Experiment 1: Comparison of Model

To validate the model’s effectiveness, this experiment introduces two distinct models for the purpose of comparative analysis. The first model assumes that MDVOMR can only be performed by SOVs under unchanged conditions, denoted as MDVOMR_B and compared with the proposed model denoted as MDVOMR. Following this development, the RPSO algorithm is applied to solve both MDVOMR and MDVOMR_B. The results are presented in Table 2 and Table 3.

As can be seen in Table 2 and Table 3, it is evident that the MDVOMR model outperforms MDVOMR_B in terms of both optimal and average solution quality, showcasing its superior comprehensive performance. This outcome compellingly underscores the enhanced operational and maintenance efficiency of the wind farm when the SOV incorporates the DV. The integration of the DV leads to a reduction in maintenance costs, thereby optimizing the wind farm’s operational effectiveness. Despite the fact that the introduction of the DV incurs additional expenses, the overall impact is a decrease in both the SOV’s operational costs and the costs associated with electricity loss. Compared with MDVOMR_B, MDVOMR incurred an additional cost of CNY 3107.2 for the DV but reduced power generation losses by CNY 1107.7, saved CNY 25,132.9 in SOV costs, and decreased the total downtime by 0.823383 h for the two turbines that required corrective maintenance. This reduction is quantified by a notable decrease in MDVOMR’s total expenses, amounting to a reduction for MDVOMR of CNY 23,133.4. Consequently, this results in significant savings of 28.85% in operational maintenance costs. The average running time for solving MDVOMR is 9.751 s, while for MDVOMR_B, it is 9.210 s. The similar solving times of the two algorithms indicate the soundness of the algorithm design; specifically, the computational workload increases only marginally when incorporating the DV sub-route. The computational complexities of solving MDVOMR and MDVOMR_B are identical.

**Figure 7:**
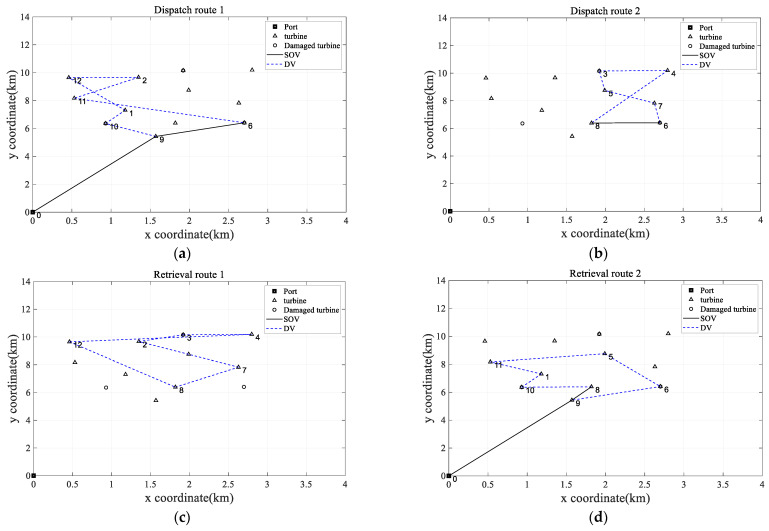
The optimal solution of MDVOMR. (**a**) The first segment of the dispatch route for the optimal solution of MDVOMR, featuring critical points at turbines 6 and 10. (**b**) The second segment of the dispatch route for the optimal solution of MDVOMR. (**c**) The first segment of the retrieval route for the optimal solution of MDVOMR. (**d**) The second segment of the retrieval route for the optimal solution of MDVOMR.

**Figure 8:**
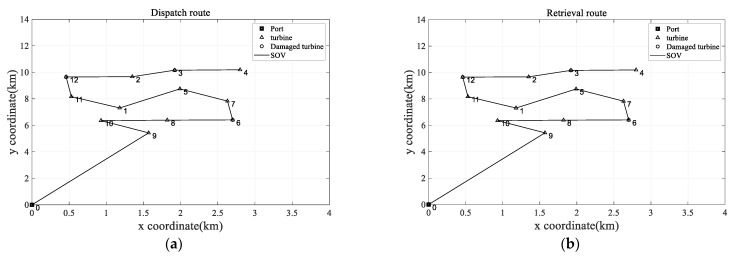
Optimal solution of MDVOMR_B. (**a**) Dispatch route for optimal solution of MDVOMR_B. (**b**) Retrieval route for optimal solution of MDVOMR_B.

For a visual representation of these optimizations, the optimal scheduling diagrams for MDVOMR and MDVOMR_B are respectively illustrated in Figure 7 and Figure 8. The specific operational maintenance scheduling routes for the MDVOMR plan are exhaustively recorded in Table 4. In this table, the “Specific Operational Maintenance Scheduling Route” section clearly marks each node’s key information in the Z(V, Y, X) format: Z represents the node number, V the time the vessel arrives at the node (in hours), Y the time the vessel leaves the node (in hours), and X the completion time of the maintenance/repair activity at the node (in hours), with the time at node 0 defined as zero. In the description of the retrieval routes, the format is simplified to Z(V, Y), including only arrival and departure times, to facilitate understanding and operation.

It is noteworthy that route crossings occur in Figure 7a,b. Why the route crossings occur is because the required maintenance time for WTs varies. Although some WTs are located near others and prioritizing their access may result in crossovers, the longer maintenance time required for these turbines justifies the priority given to them. By attending to them first, we can prioritize the completion of maintenance work, thereby facilitating a more efficient recovery route. Therefore, crossover is permitted and beneficial. To better illustrate the situation of crossover, the following section will provide a detailed description of the entire maintenance process. Specifically, operational maintenance information is as shown in Table 4.

According to Table 4, an analysis of the reasons for route crossings reveals the following: In the scenario in Figure 7a, turbine 10 requires urgent repairs and thus is prioritized. Turbine 2 needs 3 h of maintenance, and turbines 11 and 12 each require 4 h. However, in the retrieval route, prioritizing the maintenance of turbines 2 and 12 eliminates route crossings, ensuring the smooth execution of the subsequent retrieval plan. The strategy thus prioritizes maintenance at turbine 12 before moving to turbine 11. In the scenario in Figure 7b, after dispatching turbine 7, the route first leads to turbine 5, which has a 4 h maintenance window, compared to turbine 4′s 3 h maintenance duration. This priority causes route crossings to ensure the smooth execution of later retrieval plans. Additionally, as indicated by Table 4, each segment of the route adheres to endurance and load constraints. Therefore, the algorithm proposed in this paper effectively resolves MDVOMR.

#### 4.2.2. Experiment 2: Comparison of Algorithms

**Figure 9:**
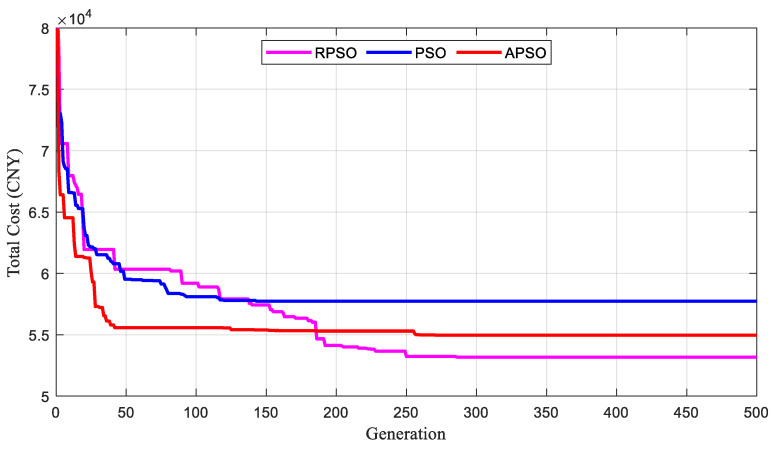
Iterative graphs of the two algorithms achieving the optimal solution.

To validate the optimization performance of the algorithm presented in this paper, three methods were employed to solve the proposed MDVOMR model, and the results were compared. The first method is the traditional PSO, the second is APSO [35], and the third is RPSO. Figure 9 illustrates the iterative processes of these algorithms in achieving optimal solutions.

**Figure 10:**
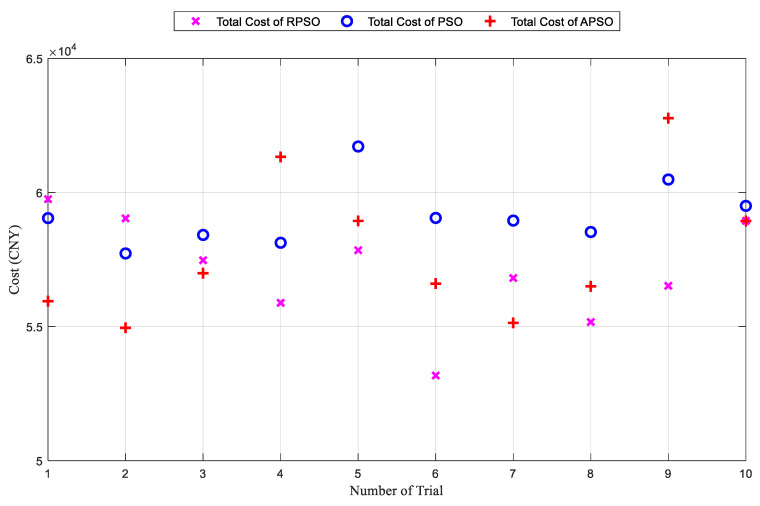
Results of ten independent experiments.

As shown in Figure 9, RPSO significantly outperforms PSO and APSO in terms of convergence. Specifically, the solutions found by RPSO and PSO stabilized at generations 250 and 150, respectively. Although APSO converges rapidly in the initial phase and can find a relatively optimal solution within fewer than 50 generations, it eventually falls into a local optimum. The results of ten independent experiments are presented in Figure 10.

Figure 10 shows that the solutions obtained by RPSO consistently outperformed those obtained by PSO in 10 independent trials. Although APSO demonstrated varying strengths compared to RPSO in 10 independent simulations, it exhibited a larger variance, with both its optimal and average solutions being inferior to those of RPSO. Further, APSO produced the worst solution among the three algorithms tested. The best solutions were achieved by RPSO. Detailed simulation data are presented in Table 5.

According to Table 5, RPSO outperforms both PSO and APSO in terms of both the optimal and average solutions. The best result from RPSO showed cost savings of CNY 4553.3 and CNY 1781.3 compared to PSO and APSO, respectively, indicating a notable optimization rate. Meanwhile, the average result from RPSO saved CNY 2093.9 compared to PSO and CNY 750.2 compared to APSO. These findings demonstrate that RPSO offers greater stability than both the traditional PSO algorithm and the APSO algorithm. Given the need to comprehensively consider various costs, the turbine downtimes for the solutions of the three algorithms are very similar. In terms of running time, the average running time of PSO is 9.018 s, while those of APSO and RPSO are 14.178 s and 9.751 s, respectively. Although APSO outperforms PSO in terms of effectiveness, it requires significantly more iteration time. In contrast, RPSO achieves better results with only a slight increase in running time, indicating that the computational workload of RPSO increases only marginally compared to PSO. Thus, it can be concluded that the computational complexities of RPSO and PSO are comparable.

## 5. Conclusions and Future Work

This paper addresses the challenging task of optimizing operations and maintenance for SOVs equipped with DVs in offshore wind farms. It proposes MDVOMR, developing a mathematical model aimed at minimizing costs. This model facilitates the joint operational and maintenance routing optimization of mother and daughter vessels within offshore wind farms, enhancing operational efficiency and reducing the downtime costs associated with maintenance and failures, thereby lowering overall operational and maintenance expenses. An RPSO is introduced to solve MDVOMR. The algorithm features an effective encoding and decoding strategy: the encoding strategy employs a vector mixed with real and binary numbers, quadruple the number of turbines, representing information for both the dispatch and retrieval phases; the decoding strategy uses ascending order sorting to determine the service sequence of turbines and a position adjustment method at the ends to choose the transport vehicle sequence for turbines, with a segmentation method determining the service routes for the SOV and DV. Based on a real-world case study involving twelve points in an offshore wind farm, this paper conducts comparative experiments to validate the effectiveness of the proposed model and algorithm; it also compares algorithms to demonstrate that RPSO surpasses traditional PSO and APSO in convergence speed, solution quality, and stability. Future research directions include expanding and improving the models and methods proposed in this paper to better suit operational or planned offshore wind farms, exploring PSO to enhance adaptability and stability across various scenarios, optimizing our algorithm and performing in-depth convergence analysis, and addressing more extensive comparative studies with other published literature.

## Figures and Tables

**Table 1:** Example parameters.

Parameters	Value	Parameters	Value
Coordinate magnification factor	100.0	Docking cost per unit for DV (CNY$\cdot h^{-1}$)	200
Turbine electricity loss cost (CNY$\cdot h^{-1}$)	1400	Maximum load capacity of DV (kg)	3600
SOV cruising speed ($\mathrm{km}\cdot h^{-1}$) [34]	20	Endurance of DV (km)	300
Cost per unit of travel for SOV (CNY$\cdot h^{-1}$)	24,000	DV resupply time (h)	0.25
Docking cost per unit for SOV (CNY$\cdot h^{-1}$)	2400	Maximum equipment transfer time (h)	0.25
DV cruising speed ($\mathrm{km}\cdot h^{-1}$) [34]	35	Maximum equipment weight required at site (kg)	900
Cost per unit of travel for DV (CNY$\cdot h^{-1}$)	2000		

**Table 2:** The simulation results of MDVOMR.

Simulation Index	MDVOMR					
	Total Cost /CNY	Cost of SOV /CNY	Cost of DV /CNY	EGL Cost /CNY	Downtime /h	Running Time /s
1	59,753.4	40,614.8	3117.9	16,020.7	11.443355	9.854
2	59,036.7	39,812.2	3203.8	16,020.7	11.443355	9.670
3	57,473.3	38,515.7	2979.7	15,977.9	11.128020	9.811
4	55,887.4	36,670.9	3142.7	16,073.8	11.481299	9.748
5	57,847.8	38,090.5	3142.1	16,615.2	11.867977	9.720
6	53,174.9	34,102.7	2847.7	16,224.5	11.588952	9.784
7	56,810.2	37,585.4	3204.1	16,020.7	11.443355	9.615
8	55,169.7	36,031.1	3117.9	16,020.7	11.443355	9.766
9	56,519.7	37,407.4	3091.6	16,020.7	11.443355	9.755
10	58,944.3	39,699.0	3224.6	16,020.7	11.443355	9.785
Average solution	57,061.8	37,853.0	3107.2	16,101.6	11.472638	9.751

**Table 3:** The simulation results of MDVOMR_B.

Simulation Index	MDVOMR_B				
	Total Cost /CNY	Cost of SOV /CNY	Electricity Loss Cost /CNY	Downtime /h	Running Time /s
1	80,509.8	63,285.1	17,224.7	12.303359	9.170
2	79,648.2	62,423.5	17,224.7	12.303359	9.144
3	84,507.8	67,283.1	17,224.7	12.303359	9.288
4	79,648.2	62,423.5	17,224.7	12.303359	9.146
5	79,584.1	62,359.4	17,224.7	12.303359	9.200
6	79,162.3	61,989.0	17,173.3	12.266668	9.240
7	79,532.7	62,359.4	17,173.3	12.266668	9.194
8	79,584.1	62,359.4	17,224.7	12.303359	9.325
9	79,820.3	62,647.0	17,173.3	12.266668	9.218
10	79,954.5	62,729.8	17,224.7	12.303359	9.176
Average solution	80,195.2	62,985.9	17,209.3	12.296021	9.210

**Table 4:** Detailed information on routes for SOV/DVs in optimal solution of MDVOMR.

Total Cost (CNY)	Route Number		Specific Service Information		Load Capacity (kg)	Travel Distance (km)
65,808.5	Dispatch Route	1	SOV Route	0-> 9 (0.282621, 0.532621, 3.53262)	400	5.6524
		2	DV Route	9 -> 10 (0.564876, 0.731543, 5.73154) -> 1 (0.75961, 0.954054, 4.95405) -> 12 (1.024, 1.21845, 5.21845) -> 2 (1.24388, 1.43833, 4.43833) -> 11 (1.48717, 1.70939, 5.70939) -> 6	3500	9.7620
			SOV Route	9 -> 6 (0.607409, 0.857409, 5.85741)	900	1.4958
		3	DV Route	6 -> 7 (2.07984, 2.32984, 4.32984) -> 5 (2.36186, 2.52853, 6.52853) -> 3 (2.56887, 2.6522, 4.6522) -> 4 (2.67736, 2.92736, 4.92736) -> 8	2700	8.7589
			SOV Route	6-> 8 (2.08323, 2.33323, 4.33323)	500	0.8802
	Retrieval Route	1	DV Route	8 -> 7 (3.33669, 4.57984) -> 2 (4.64388, 4.83833) -> 3 (4.8598, 4.94314) -> 4 (4.9683, 5.2183) -> 12 (5.28691, 5.48135) -> 8	3600	11.4596
			SOV Route	8 -> 8 (4.10522, 6.04276)	500	0
		2	DV Route	8 -> 10 (5.85772, 6.02439) -> 1 (6.05245, 6.2469) -> 11 (6.2777, 6.49992) -> 5 (6.54481, 6.71147) -> 6 (6.78134, 7.03134) -> 9	3600	8.4630
			SOV Route	8 -> 9 (5.88188, 7.32408)	400	0.9920
		3	SOV Route	9 -> 0		5.6524

**Table 5:** Statistical results.

Solution Type	Algorithm	Total Cost/CNY	Cost for SOV/CNY	Cost for DV/CNY	EGL Cost/CNY	Downtime /h	Running Time/s
Best Solution	PSO	57,728.2	38,395.1	3312.4	16,020.7	11.443355	9.048
	APSO	54,956.2	35,825.5	2906.2	16,224.5	11.588952	14.310
	RPSO	53,174.9	34,102.7	2847.7	16,224.5	11.588952	9.784
Average Solution	PSO	59,155.7	39,983.0	3098.7	16,074.0	11.481458	9.018
	APSO	57,812.1	38,680.6	3053.1	16,078.4	11.484555	14.178
	RPSO	57,061.8	37,853.0	3107.2	16,101.6	11.472638	9.751

## Data Availability

The data presented in this study are available on request from the corresponding author.
